# Seasonal and Climatic Influences on Catecholamine Metabolite Levels in Patients With and Without Pheochromocytoma–Paraganglioma

**DOI:** 10.3390/diagnostics16040588

**Published:** 2026-02-15

**Authors:** Sevgül Fakı, Abbas Ali Tam, Didem Özdemir, Pervin Demir, Salim Neşelioğlu, Gülsüm Karaahmetli, Feride Pınar Altay, Oya Topaloğlu, Reyhan Ersoy, Bekir Çakır

**Affiliations:** 1 Department of Endocrinology and Metabolism, Ankara Bilkent City Hospital, Ankara 06800, Turkey; gulsumgedik85@gmail.com (G.K.); fpaltay@gmail.com (F.P.A.); 2 Department of Endocrinology and Metabolism, Faculty of Medicine, Ankara Yildirim Beyazit University, Ankara 06800, Turkey; endoali@hotmail.com (A.A.T.); sendidem2002@yahoo.com (D.Ö.); oyasude@gmail.com (O.T.); reyhanersoy@hotmail.com (R.E.); drcakir@gmail.com (B.Ç.); 3 Department of Biostatistics and Medical Informatics, Faculty of Medicine, Ankara Yıldırım Beyazıt University, Ankara 06800, Turkey; pervin.demr@gmail.com; 4 Department of Medical Biochemistry, Faculty of Medicine, Ankara Yildirim Beyazit University, Ankara 06800, Turkey; salim_neselioglu@hotmail.com

**Keywords:** catecholamine metabolite levels, seasonal variation, climatic factors

## Abstract

**Background/Objective:** Pheochromocytomas and paragangliomas (PPGL) lead to marked catecholamine metabolite elevations, whereas the causes of mild increases remain unclear. We aimed to evaluate seasonal and climatic variation in plasma and urinary catecholamine metabolites in both PPGL and non-PPGL patients. **Methods:** We retrospectively reviewed adult patients who underwent plasma and/or 24 h urinary catecholamine metabolite testing at Ankara Bilkent City Hospital between February 2019 and May 2023. Using a big-data approach, we examined the relationship between catecholamine metabolite levels and monthly and seasonal climatic changes. **Results:** In non-PPGL patients, plasma metanephrine levels were significantly higher in autumn (*p* = 0.009) and winter (*p* = 0.027), and both plasma metanephrine (*p* < 0.001) and plasma normetanephrine (*p* = 0.003) showed transformed rank means in February that were elevated relative to the overall mean. Temperature, daylight duration, and humidity were the strongest predictors in the LASSO models. In contrast, catecholamine metabolite levels in the PPGL group showed no significant monthly or seasonal variation (all *p* > 0.05). However, when values exceeding the reference limits were examined, a significantly higher proportion of elevated plasma metanephrine measurements was observed during autumn (*p* = 0.005), whereas plasma normetanephrine elevations were most prominent during winter (*p* = 0.002). **Conclusions:** Catecholamine metabolite levels show notable variability in non-PPGL patients, which cannot be explained by seasonal comparisons alone. Monthly patterns and environmental factors—such as temperature, humidity, and photoperiod—should be considered when interpreting mild or borderline elevations to reduce false-positive results. In PPGL patients, seasonal variability was modest; however, plasma catecholamine metabolite testing showed higher diagnostic sensitivity during colder months.

## 1. Introduction

Pheochromocytomas (PCCs) and paragangliomas (PGLs) are rare neuroendocrine tumors originating from chromaffin cells. While pheochromocytomas arise from the adrenal medulla, paragangliomas originate from extra-adrenal paraganglionic tissue. Paragangliomas are therefore sometimes referred to as extra-adrenal pheochromocytomas. Despite their different anatomical locations, both tumor types are associated with significant morbidity due to excessive catecholamine secretion [1].

Collectively, pheochromocytomas and paragangliomas are referred to as pheochromocytoma–paraganglioma tumors (PPGL), a term widely used in the literature and current clinical guidelines to describe this biologically related tumor spectrum [2,3].

The incidence of PPGL ranges between 2 and 8 per million, with a prevalence between 1:2500 and 1:6500. They are most commonly diagnosed between the third and fifth decades of life. Of all PPGL, 80–85% are PCCs and 15–20% are PGLs [3,4,5]. While a large proportion of these tumors are sporadic, up to 40% of patients with PPGL harbor disease-specific germline mutations. The prevalence of PPGL among individuals carrying a germline mutation in susceptibility genes is estimated to be around 50% [6,7].

The clinical presentation of PPGL is highly variable, as their signs and symptoms are nonspecific, often leading to delayed diagnosis. The manifestations depend on the predominant catecholamine secreted, the tumor’s capacity for secretion, and its anatomical location. Epinephrine-secreting PCCs typically present with paroxysmal hypertension, palpitations, syncope, anxiety, and hyperglycemia, whereas norepinephrine-secreting PPGL more often present with headaches, sweating, and persistent hypertension. Rarely, pure dopamine-secreting tumors may present with hypotension [8,9].

Although PPGL can be detected during evaluation for symptoms suggestive of catecholamine excess or through screening in hereditary syndromes, the majority are discovered incidentally [10]. Diagnostic confirmation relies on demonstrating elevated levels of catecholamines or their metabolites in plasma or 24 h urine (24 h urine) urine. In cases of modest elevations, additional evaluations—including repeat sampling after excluding interfering conditions, medications, and dietary factors, as well as performing provocative tests such as the clonidine suppression test—may help distinguish true hypersecretion from false positives [3,11]. Nonetheless, false-positive results remain a major challenge.

Several studies have demonstrated that preanalytical and physiological factors—including patient posture during sampling, seasonal temperature variations, medications, venipuncture-related stress, and physical exercise—may lead to elevations in plasma metabolites, thereby increasing the risk of false-positive results [12,13].

In this study, we aimed to investigate whether catecholamine metabolite levels exhibit seasonal variation in both PPGL and non-PPGL patients. We also evaluated additional seasonal factors, including sunshine duration, daylight exposure, humidity, and atmospheric pressure, to examine their possible effects on catecholamine metabolite levels.

## 2. Materials and Methods

The medical records of patients evaluated in Ankara Bilkent City Hospital Endocrinology and Metabolism Diseases outpatient clinic between February 2019 and May 2023 were reviewed retrospectively for the study. All patients over the age of 18 who had catecholamine levels measured were included in the study. Catecholamine measurements were performed based on standard clinical indications such as paroxysmal hypertension, the typical adrenergic symptom triad, adrenal incidentaloma, resistant hypertension, and clinical suspicion of pheochromocytoma. Patients who underwent surgery in our hospital and were diagnosed with PPGL based on pathology results were identified. For the purposes of this study, PPGL was defined as patients who had a histopathological diagnosis of pheochromocytoma or paraganglioma. Non-PPGL was defined as patients who did not have a histopathological diagnosis consistent with PPGL.

Blood samples for free fractionated plasma metanephrine and normetanephrine were taken between 08:00 a.m. and 12:00 p.m. without instructions that patients rest supine. Potentially interfering medications and dietary factors were discontinued at least three days before 24 h urine collection. Accordingly, in most cases, blood samples were obtained after dietary preparation had been initiated; however, strict confirmation of dietary adherence at the exact time of blood sampling was not feasible in all patients due to the retrospective study design. A 24 h urine metanephrine and normetanephrine collection was performed with the addition of hydrochloric acid as a preservative, and the specimens were delivered to the laboratory the following morning upon completion of collection. In patients with biochemical elevations of less than two-fold, the tests were repeated. Plasma and 24 h urine metanephrines, and normetanephrine were measured by Liquid Chromatography with tandem mass spectrometry (LC-MS/MS) techniques.

The reference limit for plasma metanephrine was <90 pg/mL, plasma normetanephrine between February 2019 and February 2022 was <200 pg/mL, and after February 2022 was <180 pg/mL. The reference limit for 24 h urine metanephrines vary between 30 and 180 µg/24 h for women aged 17–120 years, and between 44 and 261 µg/24 h for men aged 17–120 years.

Age-specific ranges of 24 h urine normetanephrine vary as follows: 103–390 µg/24 h for individuals aged 17–29 years, 111–419 µg/24 h for those aged 29–39 years, 119–451 µg/24 h for those aged 39–49 years, 128–484 µg/24 h for those aged 49–59 years, 138–521 µg/24 h for those aged 59–69 years, and 148–560 µg/24 h for individuals aged 69–120 years.

Ankara, where the study was conducted, is at an altitude of 891 m, at 39.9727′ latitude and 32.8637′ longitude, and is a city where the temperature drops below 0 °C degrees in winter and approaches 40 °C degrees in summer. For this province, the monthly average tempera ture (°C) (Mat), daily minimum temperature average (°C) (Dminta), minimum recorded temperature within the month (°C) (Minrtm), daily maximum temperature average (°C) (Dmaxta), maximum recorded temperature within the month (°C) (Maxrtm), monthly average air pressure (hPa) (Maap), monthly average relative humidity (%) (Marh), monthly average wind speed (m/s) (Maws), daily total sunshine duration average for the month (hours) (Dtsdam), minimum day duration (hours:minutes) (Mindd), and maximum day duration (hours:minutes) (Maxdd) were obtained from the Turkish State Meteorological Service. Then, the relationships between monthly and seasonal climate events and plasma 24 h urine metanephrine and normetanephrine were analyzed.

The months of March, April, and May represented “Spring,” June, July, and August represented “Summer,” September, October, and November represented “Autumn,” and December, January, and February represented “Winter”.

This study was approved by the Ankara Bilkent City Hospital Clinical Research Ethics Committee on 11 October 2023 (E-Kurul 23-23-4706), and was conducted in accordance with the Declaration of Helsinki.

## 3. Results

### 3.1. Statistical Analysis

All statistical analyses were performed using R software (version 4.4.1; R Foundation for Statistical Computing, Vienna, Austria) and SPSS software (version 21.0; IBM Corp., Armonk, NY, USA). For all analyses, a two-tailed *p*-value < 0.05 was considered statistically significant.

The distribution of the quantitative variables was assessed using the Shapiro–Wilk’s test and graphical methods such as histograms. Quantitative variables were summarized as mean ± standard deviation or median (25th–75th percentiles) and qualitative variables as frequency (percentage). The concentration values exhibited a right-skewed distribution; therefore, a base-10 logarithmic transformation was applied to normalize the data. This transformation also rendered values unitless. For clinical interpretability, percent changes were also presented based on raw values; however, all statistical analyses were performed on log-transformed data. Comparisons of concentration values across months or seasons were performed using the Kruskal–Wallis nonparametric analysis of variance test. When a significant difference was detected, the stepwise step-down multiple comparison procedure was applied. Additionally, analyses of covariance (ANCOVA) were conducted to adjust for potential confounding effects of age and sex.

The Analysis of Means (ANOM), which compares the mean transformed rank of each group with the overall mean transformed rank, was applied to evaluate seasonal and monthly differences in transformed metanephrine values using the ANOM package in (R version 4.3.2) [14]. The transformed rank values were calculated as Φ^−1^[0.5 + (ranked value/(2n + 1))]) [15]. A result was considered statistically significant when the mean transformed rank exceeded the upper decision limit (UDL) or fell below the lower decision limit (LDL).

To minimize the potential effects of multicollinearity among predictor variables and enhance the predictive performance of the model, a Least Absolute Shrinkage and Selection Operator (LASSO) regression approach was implemented. This method applies a penalty to the absolute size of the regression coefficients, thereby constraining their magnitude and reducing model overfitting. The analysis was carried out in R using the glmnet (version 4.1-8)and caret (version 6.0-94) packages [16,17]. Unlike conventional linear regression, LASSO does not yield *p*-values for individual coefficients because it does not perform traditional hypothesis testing for variable significance. Instead, the relative magnitude of each coefficient represents the strength of its association with the dependent variable. To account for periodic fluctuations, the final model incorporated a cosinor-based approach, which models seasonal variation using trigonometric terms, including both cosine (cos) and sine (sin) components.

### 3.2. Analyses in Non-PPGL Patients

In non-PPGL patients, the study included 4408 individuals (36.9% male, 63.1% female). The mean age of males was 51.21 ± 15.31 years (median 53, range 18–90), while the mean age of females was 52.59 ± 13.69 years (median 54, range 18–91) (*p*-value: 0.069). The number of available results differed among tests: urinary metanephrine (n = 3938), plasma metanephrine (n = 4204), urinary normetanephrine (n = 3946), and plasma normetanephrine (n = 4205), as not all participants underwent all tests (Table 1).

***Monthly and seasonal variations in metanephrine and normetanephrine concentrations in non-PPGL patients.***

**Figure 1:**
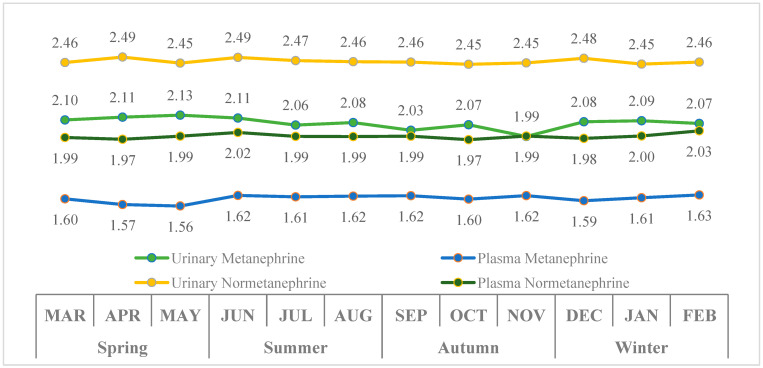
Monthly and seasonal variations in log-transformed urinary and plasma metanephrine and normetanephrine levels in non-PPGL patients.

Figure 1 illustrates the monthly and seasonal variations in urinary and plasma metanephrine and normetanephrine levels. The largest monthly variation was observed in urinary metanephrine (26.5% in raw data; 6.3% on the logarithmic scale), followed by plasma metanephrine (14.8% in raw data; 4.3% on the logarithmic scale). Variability in urinary and plasma normetanephrine was lower, at 10.0% and 12.0% in raw data (1.8% and 2.7% on the logarithmic scale), respectively.

Significant differences were observed in urinary metanephrine, plasma metanephrine, and plasma normetanephrine across months (all *p* < 0.001), whereas urinary normetanephrine did not differ significantly (*p* = 0.182). Urinary metanephrine was lowest in November, and plasma metanephrine was lower in April and May compared with other months. Seasonal comparisons revealed similar patterns, with significant differences in urinary metanephrine, plasma metanephrine, and plasma normetanephrine (all *p* < 0.001), except for urinary normetanephrine (*p* = 0.226). Post hoc analysis indicated that urinary metanephrine was lower in autumn than other seasons (Autumn < Winter = Summer = Spring) and plasma normetanephrine levels were lower in spring and autumn than in summer and winter (Spring = Autumn < Summer = Winter).

After controlling for age and gender in ANCOVA analyses, significant differences in urinary and plasma metanephrine and plasma normetanephrine levels were observed across both months and seasons, consistent with the unadjusted analyses. Urinary normetanephrine did not differ significantly between months (*p* = 0.115) or seasons (*p* = 0.100), whereas the other variables remained significantly different (all *p* < 0.001).

***Transformed rank mean comparison in each month and season for metanephrine and normetanephrine levels in non-PPGL patients*.**

**Figure 2:**
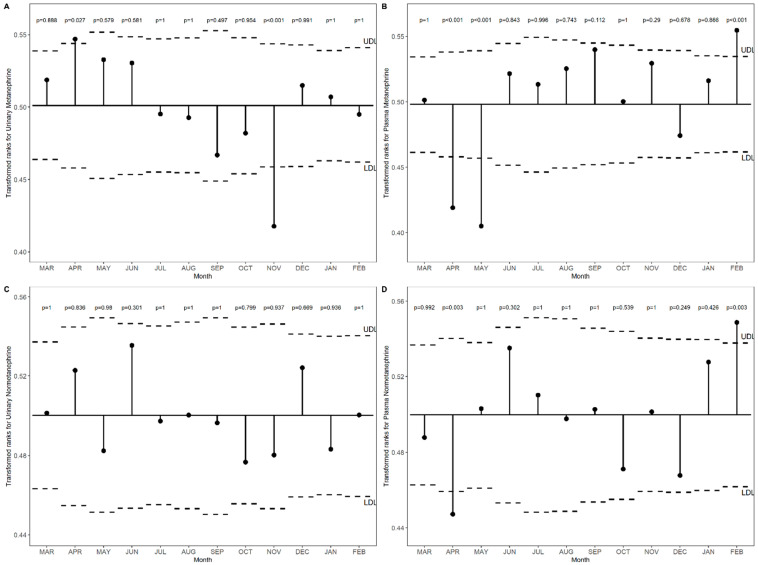
(**A**–**D**). Transformed rank means of urinary and plasma metanephrine and normetanephrine across months compared with the overall mean in non-PPGL patients.

Figure 2A–D illustrates the transformed rank means of catecholamines metabolites levels across months compared with the overall mean in non-PPGL patients. Urinary metanephrine was significantly lower in November and near the upper limit in April (*p* < 0.001). Plasma metanephrine was reduced in April and May and elevated in February (*p* < 0.001). Urinary normetanephrine remained within the defined limits throughout all months (all *p* > 0.05), whereas plasma normetanephrine decreased in April and increased in February (*p* = 0.003).

**Figure 3:**
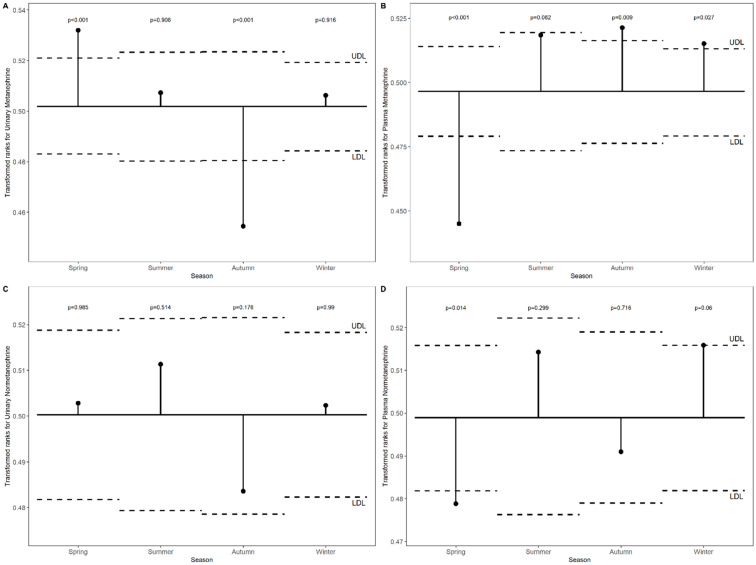
(**A**–**D**). Transformed rank means of urinary and plasma metanephrine and normetanephrine across seasons compared with the overall mean in non-PPGL patients.

Figure 3A–D shows the transformed rank means of catecholamines metabolites levels across seasons compared with the overall mean in non-PPGL patients. Urinary metanephrine was significantly elevated in Spring and decreased in Autumn (*p* < 0.001). Plasma metanephrine was reduced in Spring (*p* < 0.001) and slightly increased in Autumn (*p* = 0.009) and Winter (*p* = 0.027). Urinary normetanephrine remained within the overall mean range for all seasons (*p* > 0.05), whereas plasma normetanephrine was significantly lower in Spring (*p* = 0.014).

**Figure 4:**
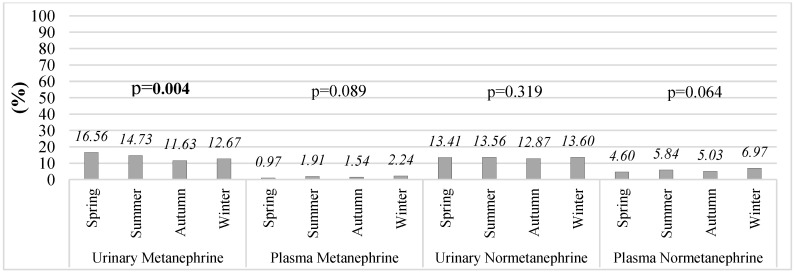
Seasonal changes in the percentage of subjects exceeding reference limits for urinary and plasma metanephrines and normetanephrines in non-PPGL patients.

When the proportions of subjects exceeding the reference limits were evaluated seasonally, a significant seasonal difference was observed only in urinary metanephrine levels (*p* = 0.004) (Figure 4). Specifically, the proportion of individuals with elevated urinary metanephrine in spring (16.56%) was significantly higher than that observed in autumn (11.63%).

**Monthly/seasonal distribution of average air temperature and log-transformed urinary and plasma *metanephrine and normetanephrine* levels in non-PPGL patients** **.**

**Figure 5:**
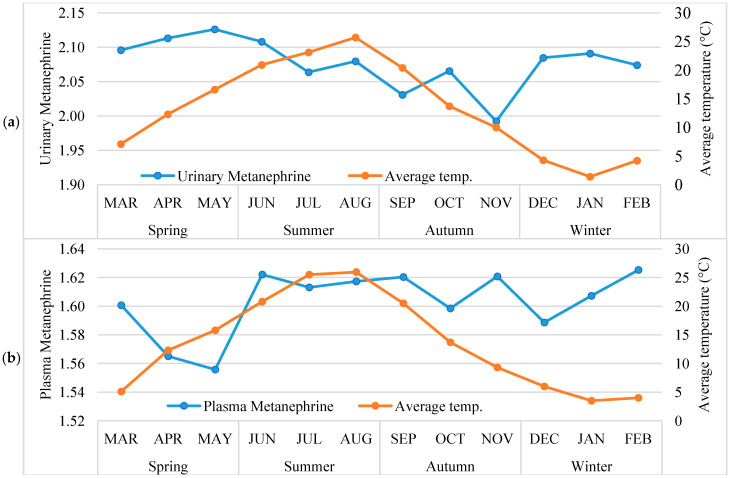

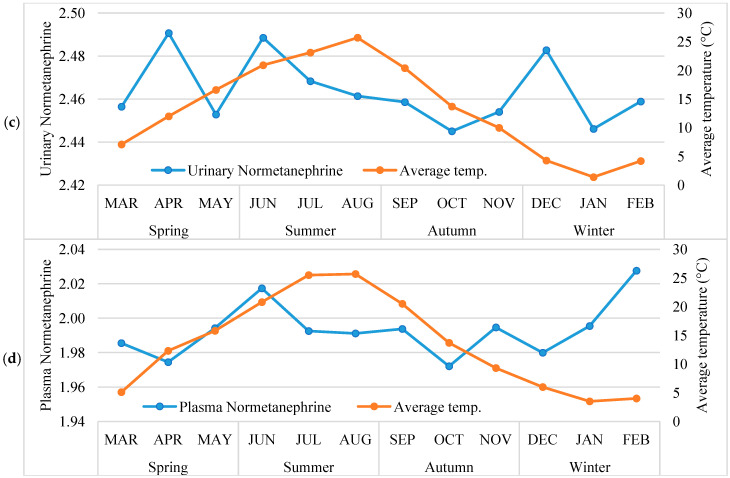
(**a**–**d**). The distribution of monthly average air temperature (°C) according to months/season and the median values of log-transformed urinary, plasma *metanephrine and normetanephrine* levels in non-PPGL patients.

Based on Figure 5, urinary and plasma metanephrine and normetanephrine levels remained generally stable throughout the year, with only minor fluctuations observed. The lowest urinary metanephrine value was seen in November (1.99), while no significant seasonal changes were detected in the other parameters. No clear correlation was observed between average temperature variations and metanephrine levels.

**Examination of the effects of methodological data on metanephrine concentrations.**

The LASSO regression model was used to examine the effects of meteorological factors on urinary and plasma metanephrine and normetanephrine concentrations, controlling for age and gender and incorporating cyclical components to account for monthly and seasonal variations (Table 2). Due to the penalization structure of the LASSO regression model, traditional inferential statistics such as *p*-values are not provided. Instead, model performance and explanatory capacity were evaluated using RMSEA and R^2^ values. The relatively high RMSEA and low R^2^ values observed across models indicate limited model fit and low explanatory power, suggesting that the models are more suitable for identifying variable importance rather than establishing statistical significance.

In the monthly model, urinary metanephrine was mainly influenced by maximum temperature (Dmta, 100%), minimum daylight duration (Mindd, 91.8%), and maximum daylight duration (Maxdd, 79.3%). Plasma metanephrine showed strong cyclical effects, driven by the sine (100%) and cosine (80.2%) components and maximum daylight duration (97.3%). For urinary normetanephrine, minimum daylight duration (100%), maximum daylight duration (85.7%), and maximum temperature (39.8%) were key predictors, while plasma normetanephrine was primarily shaped by the sine (100%) and cosine (69.4%) components, minimum daylight duration (86.8%) and mean air temperature (Mat, 62.06%).

In the seasonal model, urinary metanephrine was most affected by maximum temperature (Dmta, 100%), relative humidity (Marh, 72.9%), and atmospheric pressure difference (Dtsdam, 67.8%). Plasma metanephrine was driven by the cosine component (100%), relative humidity (62.9%), and mean air pressure (Maap, 21.7%). Urinary normetanephrine was influenced mainly by pressure difference (Dtsdam, 100%), humidity (78.1%), and temperature (Dmta, 39.9%), while plasma normetanephrine was associated with mean air temperature (Mat, 100%), maximum temperature (Dmta, 60.7%), and minimum temperature (Dminta, 27.4%).

Overall, both models emphasize the significant roles of temperature, daylight duration, humidity, and air pressure, with the sine and cosine components underscoring the cyclical (monthly and seasonal) variations in metanephrine and normetanephrine levels.

### 3.3. Analyses in PPGL Patients

Among the 90 patients who were clinically, biochemically, and radiologically diagnosed with PPGL, histopathological results were not accessible in 5 cases (5.6%). Among patients with available histopathology, diagnoses included pheochromocytoma in 71 patients (78.9%), paraganglioma in 9 patients (10.0%), malignant pheochromocytoma in 1 patient (1.1%), and adrenal medullary hyperplasia in 5 patients (5.6%). For the final analysis, only patients with pheochromocytoma and paraganglioma were included, resulting in a study cohort of 80 patients.

The mean age of the study population was 51.01 ± 13.78 years, and 51.3% of the patients were female. No significant differences in age or sex were observed between the pheochromocytoma and paraganglioma groups (*p* = 0.145 and *p* = 0.734, respectively).

Genetic testing was requested based on clinical suspicion. MEN2 analysis was requested in 10 patients, familial cancer panel testing in 2 patients, 1 patient had a confirmed diagnosis of von Hippel–Lindau (VHL) syndrome, and two patients had a documented history of neurofibromatosis (NF). However, genetic test results were not available for all requested analyses due to ethical and data protection constraints inherent to the retrospective design. In addition, paraganglioma-associated susceptibility genes, such as *SDHB* and other *SDHx* genes, are not reimbursed under the national health insurance system (SGK SUT) and therefore could not be routinely analyzed.

Regarding tumor localization, 9 patients were diagnosed with extra-adrenal paraganglioma, while 76 patients had adrenal-origin tumors consistent with pheochromocytoma. The anatomical distribution of paragangliomas was as follows: para-aortic region anterior to the left adrenal gland (*n* = 3), cervical region adjacent to the internal carotid artery (*n* = 2), right adrenal upper pole region (*n* = 1), between the gastric corpus and pancreatic body (*n* = 1), abdominal periportal region (*n* = 1), and paravertebral region adjacent to the inferior pole of the left kidney (*n* = 1). This detailed localization information has also been added to the Materials and Methods section.

Table 3 summarizes the urinary and plasma metanephrine and normetanephrine concentrations measured at different time points across all PPGL patients identified based on clinical, biochemical, and radiological evaluation (n = 80). A total of 95–105 measurements were obtained per analyte.

**Figure 6:**
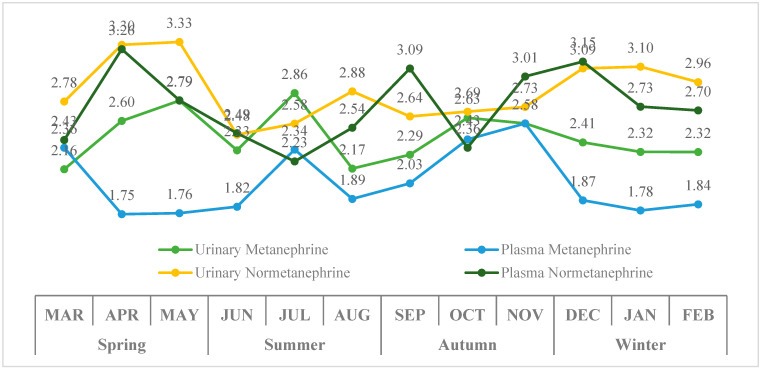
Monthly and seasonal variations in log-transformed urinary and plasma metanephrine and normetanephrine levels in PPGL patients.

In the cohort of 80 patients, urinary and plasma metanephrine and normetanephrine levels were summarized across all available measurements. Monthly variability was highest for plasma normetanephrine (90.7% log_10_; 31.6% raw), followed by urinary normetanephrine (85.9% log_10_; 25.6% raw), plasma metanephrine (85.3% log_10_; 32.3% raw), and urinary metanephrine (80.1% log_10_; 24.4% raw). No significant differences were observed across months for urinary metanephrine, plasma metanephrine, urinary normetanephrine, or plasma normetanephrine levels (all *p* > 0.05). Similarly, no significant seasonal variations were detected in any of the four measurements (all *p* > 0.05) (Figure 6).

**Figure 7:**
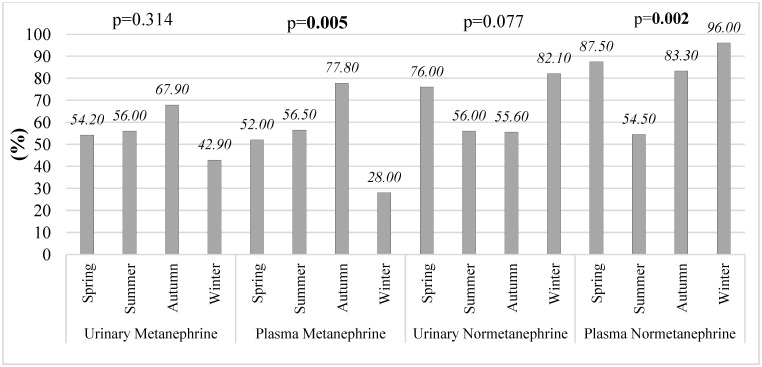
Seasonal changes in the percentage of patients exceeding reference limits for urinary and plasma metanephrines and normetanephrines in PPGL patients.

The proportion of patients with urinary metanephrine and urinary normetanephrine levels above the reference limits did not differ significantly across seasons (*p* = 0.314 and *p* = 0.077, respectively), indicating similar seasonal distribution patterns (Figure 7). In contrast, plasma metanephrine and plasma normetanephrine showed significant seasonal variation, with a higher proportion of elevated values observed in certain seasons (*p* = 0.005 and *p* = 0.002, respectively).

**Figure 8:**
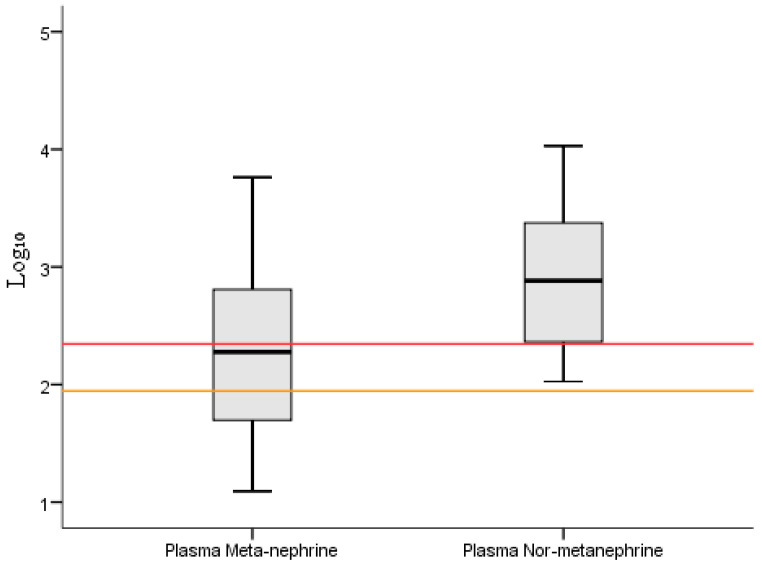
Box-and-whisker plot of plasma-free metanephrine and normetanephrine levels (log_10_ Scale) in pheochromocytoma patients (the red horizontal line indicates the upper reference limit for plasma normetanephrine, and the orange horizontal line indicates the upper reference limit for plasma metanephrine).

Figure 8 illustrates the distribution of plasma-free metanephrine and normetanephrine concentrations on a log_10_-transformed values scale. Plasma normetanephrine levels were generally higher than plasma metanephrine levels, with a right-shifted distribution and a higher median value. A larger proportion of measurements exceeded the diagnostic cut-off thresholds for normetanephrine compared with metanephrine, indicating a predominantly normetanephrine-driven biochemical profile in the study population.

**Figure 9:**
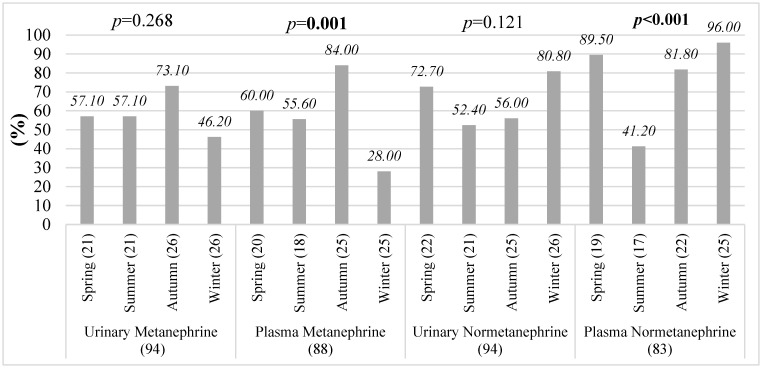
Test-based seasonal variation in plasma catecholamine metabolites results exceeding reference limits in pheochromocytoma patients.

In patients with pheochromocytoma, seasonal analyses based on the total number of plasma measurements demonstrated parameter-specific differences in the proportion of results exceeding reference limits across the four seasons (Figure 9). Urinary metanephrine and urinary normetanephrine levels did not show significant seasonal variation, with comparable proportions of measurements exceeding reference limits throughout the year (*p* = 0.268 and *p* = 0.121, respectively).

In contrast, significant seasonal variation was observed for plasma-derived catecholamine metabolites. Plasma metanephrine measurements showed the highest proportion of values exceeding the reference limits during autumn (84%; *p* < 0.05), indicating increased diagnostic sensitivity during this period. Similarly, plasma normetanephrine showed marked seasonal variation, with the highest proportion of elevated measurements observed during winter months (*p* < 0.05). Notably, during winter, plasma normetanephrine values exceeded the reference limits in up to 96% of measurements, representing the most pronounced seasonal effect among all analyzed parameters.

**Figure 10:**
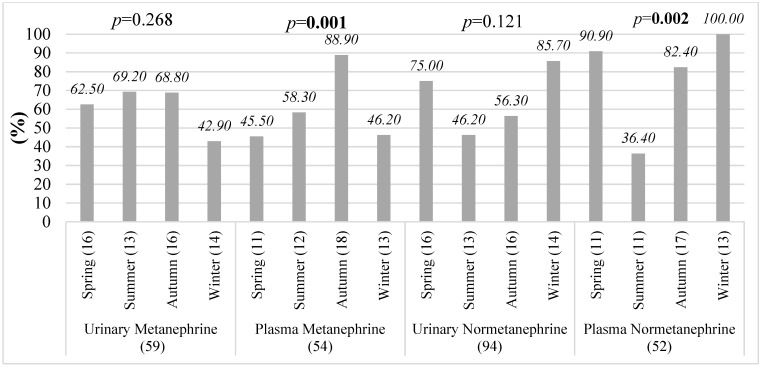
Patient-based seasonal distribution of plasma metanephrine and normetanephrine measurements exceeding reference limits in pheochromocytoma patients.

In patients with pheochromocytoma, a patient-based seasonal analysis was performed using catecholamine metabolite measurements obtained at the time of initial clinical presentation, to evaluate seasonal differences in the proportion of patients with plasma values exceeding reference limits (Figure 10).

Plasma metanephrine showed the highest proportion of patients with values exceeding reference limits during autumn, reaching approximately 89% (*p* = 0.001). In contrast, plasma normetanephrine exhibited a marked winter predominance (*p* = 0.002), with levels exceeding reference limits in nearly all patients during the winter months.

In patients with paraganglioma, seasonal analyses did not demonstrate significant differences in catecholamine metabolite levels in either test-based or patient-based analyses. Specifically, no significant seasonal variation was observed for urinary metanephrine (*p* = 0.0458 and *p* = 0.494), plasma metanephrine (*p* = 0.223 and *p* = 0.301), urinary normetanephrine (*p* = 0.458 and *p* = 0.112), or plasma normetanephrine (*p* = 0.466 and *p* = 0.549), respectively.

## 4. Discussion

Although PPGL are rare, especially during the COVID-19 pandemic, the widespread use of computed tomography (CT) markedly increased the detection of masses in likely PPGL locations. Therefore, biochemical testing became more frequent, and modest increases in plasma and urinary catecholamine metabolites posed considerable diagnostic challenges for clinicians.

Cold adaptation is mediated by coordinated neuroendocrine responses (primarily involving catecholamines such as noradrenaline and adrenaline, thyroid hormones, and cortisol), which enhance energy production by increasing sympathetic activity. İn our study, we examined both plasma and urinary catecholamine metabolites in patients with and without PPGL, thereby allowing a direct comparison of the impact of seasonal and climatic variations on catecholamine secretion in both tumor-driven and non-tumor-related physiological conditions. In the PPGL group, plasma and urinary metanephrine and normetanephrine levels exhibited varying degrees of monthly variability; however, no statistically significant differences were observed across months or between seasons for any of the four measurements. In PPGL patients, plasma metanephrine demonstrated higher diagnostic sensitivity in autumn, while plasma normetanephrine peaked during winter. This seasonal effect was particularly evident in pheochromocytoma patients, in whom diagnostic sensitivity increased markedly, reaching up to 89–100%, respectively. In contrast, among non-PPGL patients, significant differences were observed in urinary metanephrine, plasma metanephrine, and plasma normetanephrine levels across months, except for urinary normetanephrine, which did not show significant variation.

To the best of our knowledge, the first study on this subject was conducted in 1976, reporting results from a total of five years and examining urinary total metanephrine levels in 1414 patients. They observed that urinary total metanephrine excretion was significantly higher and more variable in winter compared to summer months, with mean values around 800 µg/day in winter and 300 µg/day in summer [18]. In their study, urine samples collected from four volunteer subjects, both with and without the addition of hydrochloric acid, were analyzed during the summer and winter months, and it was determined that the inter-assay variation of the assay was not responsible for the observed seasonal variation.

In our study, a total of 3938 urinary metanephrine measurements were analyzed. Both monthly and seasonal variations were observed in urinary metanephrine levels. Urinary metanephrine concentrations were significantly lower in November and near the upper limit in April, showing a significant increase during spring and a decrease in autumn. Unlike the previous study, in our investigation potentially interfering medications and dietary factors were discontinued prior to sampling, and hydrochloric acid was added during urine collection to ensure sample stability and analytical accuracy. These are interesting findings. In the seasonal model, urinary metanephrine was positively associated with maximum temperature (Dmta, 100%), whereas it showed negative associations with relative humidity (Marh, –0.145; 72.9%) and total daily sunshine duration (Dtsdam, –0.135; 67.8%). This suggests that urinary metanephrine excretion tends to increase during warmer but less humid periods, emphasizing that urinary metanephrine levels are influenced not only by temperature but also by broader climatic factors affecting autonomic regulation.

Although no significant seasonal variation was observed for urinary normetanephrine in our study, interestingly, the regression analysis revealed several noteworthy associations. Urinary normetanephrine levels increased as the minimum daylight duration decreased (Mindd, −0.113; 100%), indicating higher values during shorter winter days. In addition, relative humidity (Marh, −0.215; 78.05%) and total daily sunshine duration (Dtsdam, −0.275; 100%) were negatively correlated with urinary normetanephrine, suggesting that levels tended to rise under less humid and less sunny conditions. To our knowledge, no previous studies have specifically investigated urinary normetanephrine in relation to these environmental or climatic parameters.

Based on data collected from two tertiary referral centers, Pamporaki et al. evaluated 4052 patients, of whom 107 were diagnosed with PCC and 3945 were tumor-free. In tumor-free patients, plasma normetanephrine levels were approximately 21% higher in winter than in summer, resulting in a two-fold increase in false-positive results and a decline in diagnostic specificity from 96% to 92% [19]. Similarly, Yu and Wei analyzed 407 patients in Los Angeles and reported that plasma normetanephrine concentrations were 42.3% higher in winter compared to summer [20]. In contrast, Griffin et al., who evaluated 663 patients in the West of Ireland, found no significant seasonal variation in plasma normetanephrine, which they attributed to the narrow temperature range (14.3 °C in summer vs. 5.8 °C in winter) and persistently high humidity, suggesting that climate may exert a protective effect against seasonal fluctuations [21].

In our study, plasma normetanephrine measurements were analyzed in 4205 non-PPGL patients. Although no statistically significant difference was detected between winter and summer, plasma normetanephrine levels increased in February and decreased in April, occasionally exceeding the upper reference limit. Consistent with this, the monthly model showed that plasma normetanephrine tended to rise as daylight duration shortened (Mindd decreased) and temperature dropped (Mat negatively correlated, coef. = −0.341). These findings are in line with previous studies and suggest that a month-based evaluation may better reflect the physiological variation of plasma normetanephrine levels. It should be noted that our definition of “season” is identical to that used by Pamporaki et al. but different to the definition used by Yu and Wei (summer: July–September; autumn: October–November) [19].

Additionally, Pamporaki et al. reported that forearm warming enhanced blood flow and reduced plasma normetanephrine concentrations, indicating that ambient temperature may significantly influence catecholamine metabolite measurements [19]. Consistent with this observation, in our seasonal LASSO regression model, plasma normetanephrine levels were mainly influenced by mean air temperature (Mat, 100%), maximum temperature (Dmta, 60.7%), and minimum temperature (Dminta, 27.4%). The positive coefficient for mean air temperature indicates that plasma normetanephrine concentrations tend to increase under moderate temperature conditions, whereas extreme thermal conditions (both high and low) were associated with reduced plasma normetanephrine levels.

In contrast to previous studies by Pamporaki et al., Yu and Wei, and Griffin et al., all of which reported no significant seasonal variation in plasma metanephrine, our findings demonstrated a clear cyclical pattern. A total of 4204 plasma metanephrine measurements were analyzed, revealing significant monthly variation (14.8% in raw data; 4.3% on the logarithmic scale). Plasma metanephrine concentrations were lowest in April and May and highest in February. Seasonal comparisons further showed that plasma metanephrine was significantly reduced in spring and slightly increased in autumn and winter.

Cardiovascular and cerebrovascular epidemiology strongly supports the concept of temporal clustering of events. Large multicountry analyses involving 19 nations have shown that cardiovascular mortality is consistently higher during winter months across diverse latitudes [22]. Similarly, a national registry analysis from New Zealand demonstrated that hospitalizations for myocardial infarction and deaths from ischemic heart disease peak during winter, with event ratios approximately 1.29 compared with summer [23]. In the Shiga Stroke Registry, which included 6688 first-ever stroke events, seasonal variation was more pronounced in intracerebral hemorrhage than in ischemic stroke, with the highest incidence observed during the winter period (December–February) [24]. Moreover, in a 10-year hospital-based study from Iran encompassing 24,186 stroke cases, Bahonar et al. reported a significantly higher 28-day mortality in February compared with March (UMR 1.19; 95% CI 1.00–1.42), highlighting the value of month-specific rather than broad seasonal analyses [25].

In our non-PPGL cohort, plasma metanephrine and plasma normetanephrine levels were significantly above the overall mean in February, indicating a distinct monthly peak despite the absence of a significant winter–summer contrast in broader seasonal comparisons. This biochemical pattern may plausibly reflect late-winter increases in sympathetic drive or catecholamine turnover, mechanisms that have been proposed to contribute to the temporal clustering of adverse cardiovascular and cerebrovascular events observed in epidemiological studies. Nevertheless, our study did not assess clinical outcomes, and therefore any link between February elevations in catecholamine metabolites and cardiovascular risk remains speculative. Well-designed prospective studies are required to clarify whether late-winter fluctuations in catecholamine metabolism translate into a measurable increase in cardiovascular or cerebrovascular event risk.

In contrast to Pamporaki et al., who reported no seasonal differences in patients with pheochromocytoma (PCC) [19], Pommer et al. evaluated patients with PPGL separately according to inpatient and outpatient sampling and found that the influence of ambient temperature on plasma normetanephrine concentrations and false-positive results was significant only in outpatients [13]. Their regression analysis demonstrated that a 30 °C decrease in temperature led to a 25% increase in plasma normetanephrine levels in outpatients, whereas this effect was markedly attenuated in inpatients. The authors suggested that adaptation of the sympathetic nervous system to warm indoor conditions might contribute to lower normetanephrine levels and fewer false-positive results in inpatients. In our study, which included 80 patients with PPGL, both plasma metanephrine and plasma normetanephrine exhibited significant seasonal variation, with a higher proportion of elevated values observed in autumn (77.8%) and winter (96.0%), respectively. These findings are consistent with those of Pommer et al., suggesting that plasma normetanephrine tends to increase during colder periods.

In subgroup analyses, no significant seasonal variation was observed in patients with paraganglioma. In contrast, among patients with pheochromocytoma, clear seasonal differences were identified in plasma catecholamine metabolite measurements. During the autumn season, plasma metanephrine concentrations exceeded the upper reference limit in approximately 90% of patients, whereas plasma normetanephrine concentrations exceeded the upper reference limit in nearly 100% of patients during the winter season. Accordingly, the diagnostic sensitivity of plasma catecholamine measurements was highest during these respective seasons. Overall, our results were found to be consistent with those reported in previous studies, demonstrating higher rates of plasma catecholamine positivity—and consequently higher diagnostic sensitivity—during colder seasons, particularly for plasma normetanephrine [19].

In accordance with current guideline recommendations, screening for suspected PPGL should be performed in patients with uncontrolled or early-onset hypertension, symptoms suggestive of catecholamine excess, adrenal or paraganglionic masses in typical locations, a history of PPGL, or known genetic susceptibility. Plasma-free metanephrines or 24 h urinary fractionated metanephrines are recommended as first-line biochemical screening tests, with plasma measurements offering slightly higher sensitivity in high-risk patients. However, false-positive results are relatively common and may arise from medications, dietary factors, renal impairment, and preanalytical conditions [26]. In this clinical framework, awareness of potential seasonal influences—particularly the increased sensitivity of plasma metanephrine and normetanephrine measurements during colder months—may help clinicians interpret borderline results more cautiously and facilitate earlier diagnosis and timely management.

Taken together, the limited available literature demonstrates that seasonal variation in catecholamine metabolites is inconsistently observed across different climates and methodological settings (Table 4). Our study contributes additional data highlighting the influence of temperature, humidity, and daylight duration on catecholamine metabolism, particularly in non-tumor conditions.

## 5. Conclusions

In this retrospective study from a single tertiary endocrine center, we showed that urinary metanephrine, plasma metanephrine, and plasma normetanephrine levels are influenced not only by tumor-related hypersecretion but also by season and climatic conditions, particularly in patients without pheochromocytoma or paraganglioma. Among non-tumor patients, plasma and urinary metanephrines and plasma normetanephrine demonstrated clear monthly and seasonal patterns and were associated with ambient temperature, humidity, and daylight duration, indicating that physiological catecholamine turnover is dynamically modulated by environmental factors. These fluctuations may partly explain modest or borderline elevations and thus contribute to false-positive biochemical results in routine practice.

By contrast, in patients with pheochromocytoma or paraganglioma, catecholamine metabolite concentrations were markedly elevated and did not show clinically meaningful seasonal variation, suggesting that tumor-driven secretion largely overrides environmental influences. From a practical standpoint, our findings do not support the need for season-specific diagnostic thresholds for metanephrines in our setting; however, they underline the importance of interpreting small increases in the context of sampling conditions, season, and coexisting physiological stressors, especially in patients without a confirmed tumor.

This study has several limitations. First, its single-center, retrospective design may limit the generalizability of the findings to other populations or climatic regions. Second, blood samples for plasma metanephrines were obtained in the seated position rather than after a standardized period of supine rest, which is known to influence catecholamine metabolite concentrations and may have contributed to variability in the results. Third, the study relied on routine clinical sampling rather than strictly controlled preanalytical conditions, and although interfering medications and dietary factors were minimized, unmeasured confounders cannot be fully excluded. Another limitation of this study is the lack of systematic germline genetic testing. Although genetic evaluation was initiated in several patients based on clinical indication, ethical constraints, incomplete availability of results, and reimbursement policies limited access to comprehensive genetic data, thereby restricting detailed genotype-based subgroup analyses. In addition, all measurements were analyzed together, including multiple samples collected from the same individual patients. Although a within-subjects design could offer greater statistical power than between-subjects comparisons, evaluating seasonal variation based on serial measurements from the same individual is not practical, as restructuring such data is both time-consuming and difficult to implement in routine clinical practice. Finally, as our study was retrospective, prospective studies incorporating serial measurements from the same individuals across different seasons are needed to more clearly delineate true within-subject seasonal variation in catecholamine metabolites.

## Figures and Tables

**Table 1:** Descriptive statistics of urinary and plasma metanephrine and normetanephrine concentrations, with original units and log_10_-transformed values in non-PPGL patients.

Concentration	Mean ± SD	Median (Min–Max)	Log_10_ Mean ± SD	Log_10_ Median (Min–Max)
Urinary Metanephrine (μg/24 h)	138.80 ± 130.53	119.72 (2.40–4304.99)	2.06 ± 0.27	2.08 (0.38–3.63)
Plasma Metanephrine (pg/mL)	44.00 ± 29.05	39.95 (6.80–1228.30)	1.61 ± 0.17	1.6 (0.83–3.09)
Urinary Normetanephrine (μg/24 h)	323.67 ± 251.42	290.20 (6.96–5982.74)	2.44 ± 0.26	2.46 (0.84–3.78)
Plasma Normetanephrine (pg/mL)	113.46 ± 81.89	98.27 (4.00–2652.74)	2.01 ± 0.18	1.99 (0.60–3.42)

SD: standard deviation, min–max: minimum–maximum, log_10_: values are transformed using base-10 logarithm (log_10_) to normalize skewed distributions.

**Table 2:** The ranking of the importance of each independent variable on the dependent variable in non-PPGL patients.

	Urinary Metanephrine		Plasma Metanephrine		Urinary Normetanephrine		Plasma Normetanephrine	
**Month**	**Coef.**	**Imp. %**	**Coef.**	**Imp. %**	**Coef.**	**Imp. %**	**Coef.**	**Imp. %**
Age	−0.011	9.91	0.000	0.00	0.041	36.04	0.024	4.18
Gender	−0.047	40.82	−0.035	9.67	−0.015	13.53	−0.023	3.99
Mat	0.000	0.00	−0.015	4.24	0.000	0.00	−0.341	**62.06**
Dminta	−0.008	6.88	0.059	16.64	−0.004	3.07	0.311	**56.61**
Minrtm	−0.014	11.81	−0.025	6.98	−0.028	24.63	−0.056	10.03
Dmta	0.115	**100.00**	0.027	7.67	0.000	0.00	0.132	23.86
Maxrtm	−0.049	42.62	−0.048	13.36	0.045	**39.84**	−0.114	20.48
Maap	−0.025	22.17	0.020	5.67	−0.021	19.04	0.009	1.42
Marh	−0.040	34.92	−0.069	19.40	−0.035	30.63	−0.087	15.64
Maws	−0.019	16.54	0.021	5.82	−0.010	8.80	−0.001	0.00
Dtsdam	−0.010	8.32	−0.001	0.27	−0.032	28.69	−0.006	0.86
Maxdd	0.091	**79.25**	0.347	**97.26**	0.096	**85.68**	0.477	**86.82**
Mindd	−0.105	**91.80**	0.045	12.56	−0.113	**100.00**	0.188	34.11
Cosine component	0.066	57.34	0.286	**80.17**	0.007	6.18	0.382	**69.42**
Sine component	0.000	0.00	−0.356	**100.00**	0.000	0.00	−0.549	**100.00**
Intercept	2.057		1.605		2.437		2.011	
**Season**	**Coef.**	**Imp. %**	**Coef.**	**Imp. %**	**Coef.**	**Imp. %**	**Coef.**	**Imp. %**
Age	−0.013	5.27	0.000	0.01	0.036	13.17	0.024	0.92
Gender	−0.047	22.94	−0.034	10.07	−0.015	5.52	−0.023	0.87
Mat	0.007	2.37	0.075	22.04	0.058	20.99	2.234	**100.00**
Dminta	0.004	0.78	0.000	0.00	0.000	0.00	−0.615	**27.39**
Minrtm	−0.025	11.44	0.101	29.55	0.020	7.33	−0.048	1.96
Dmta	0.197	**100.00**	0.000	0.00	0.110	**39.93**	−1.358	**60.74**
Maxrtm	−0.089	44.60	−0.003	0.96	0.019	6.81	−0.046	1.88
Maap	−0.034	16.34	−0.074	21.67	−0.029	10.43	−0.044	1.82
Marh	−0.145	**72.98**	−0.214	**62.99**	−0.215	**78.05**	−0.259	11.44
Maws	0.002	0.00	0.023	6.71	0.031	11.36	0.050	2.08
Dtsdam	−0.135	67.77	−0.074	21.70	−0.275	**100.00**	−0.237	10.47
Maxdd	0.032	15.21	−0.010	3.02	0.083	30.18	−0.011	0.34
Mindd	−0.005	1.59	0.000	0.10	−0.066	23.89	0.030	1.19
Cosine component	0.148	74.56	0.340	**100.00**	0.184	67.05	0.273	12.08
Sine component	0.040	19.19	0.000	0.07	0.043	15.64	−0.004	0.00
Intercept	2.057		1.605		2.437		2.011	

Coef: model coefficient, Imp.: variable importance percentage derived from the model. Cosine and sine components represent the amplitude and phase of a cyclic pattern, respectively. The use of both sine and cosine terms is a common approach to model seasonality because it allows the model to consider both the amplitude (captured by the sine term) and the phase (captured by the cosine term) of the seasonal effect. Variables with coefficient values of 0.000 indicate no significant effect on the dependent variable; in LASSO regression, these predictors are shrunk to zero and excluded from the model. Mat: monthly average temperature, Dminta: daily minimum temperature average, Minrtm: minimum recorded temperature within the month, Dmta: daily maximum temperature average, Maxrtm: maximum recorded temperature within the month, Maap: monthly average air pressure (hPa), Marh: monthly average relative humidity (%), Maws: monthly average wind speed (m/s), Dtsdam: daily total sunshine duration average for the month (hours), Mindd: minimum day duration (hours:minutes), Maxdd: maximum day duration (hours:minutes).

**Table 3:** Descriptive statistics of urinary and plasma metanephrine and normetanephrine concentrations in patients (n = 80), with original units and log_10_-transformed values.

Concentration	n. of Measurement	Mean ± SD	Median (Min–Max)	Log_10_ Mean ± SD	Log_10_ Median (Min–Max)
Urinary Metanephrine (μg/24 h)	105	1127.54 ± 2981.47	284 (9; 23,234)	2.55 ± 0.59	2 (1; 4)
Plasma Metanephrine (pg/mL)	100	424.12 ± 954.29	108 (12; 5775)	2.16 ± 0.58	2 (1; 4)
Urinary Normetanephrine (μgg/24 h)	105	2133.17 ± 3673.94	774 (15; 22,687)	2.94 ± 0.59	3 (1; 4)
Plasma Normetanephrine (pg/mL)	95	1408.42 ± 1825.63	620 (41; 10,698)	2.84 ± 0.54	3 (2; 4)

SD: standard deviation, min–max: minimum–maximum, log_10_: values are transformed using base-10 logarithm (log_10_) to normalize skewed distributions.

**Table 4:** Summary of seasonal and temperature effects on plasma and urinary metanephrines in patients with PPGL and non-PPGL.

Study (n)	Seasonal Effect	Temperature Effect
Yamamoto et al., (n = 1414), [18]	Winter ↑ **Urinary** tMN (~800 µg/day) vs. summer (~300 µg/day);	Strong seasonal amplitude in non-PPGL patients
Pamporaki et al., (n = 4052), [19]	Winter ↑ PNMN ~21% in non-PCC; no effect in PCC; MN stable	NMN inversely correlated with temperature
Yu & Wei, (n = 407), [20]	Winter ↑ PNMN +42.3%; PMN stable	Effect persisted in non-PPGL patients despite mild climate in Los Angeles
Griffin et al., (n = 663), [21]	No seasonal variation in PNMN; slight PMN rise in autumn	No correlation with temperature/humidity in non-PPGL patients
Pommer et al., (n = 3147), [13]	≤13.8 °C:P NMN +8.5%; effect only in outpatients	in non-PPGL patients seasonal effect mediated via ambient temperature
Present study (PPGL)	Plasma: PMN ↑ in autumn (77.8%); PNMN ↑ in winter	NMN ↑ during colder and shorter-day periods
Present study (non-PPGL)		
└ Plasma Metanephrine	↓ in **spring** (Spring < Autumn = Winter = Summer)	↑ with moderate temperature; ↓ under extreme thermal conditions
└ Urinary Metanephrine	↓ in **autumn** (Autumn < Winter = Summer = Spring)	↑ with higher temperature; ↓ with high humidity
└ Plasma Normetanephrine	↓ in **spring and autumn** (Spring = Autumn < Summer = Winter)	Inversely correlated with temperature; ↑ in colder and shorter-day periods
└ Urinary Normetanephrine	No significant seasonal change	↓ with higher humidity and sunshine; ↑ when daylight shorter

PMN = metanephrine; PNMN = normetanephrine; tMN = total metanephrine; ↑ increase in the variable, ↓ decrease in the variable

## Data Availability

The data underlying this article are not publicly available due to patient privacy and institutional regulations but are available from the corresponding author on reasonable request.

## Abbreviations

CT	Computed Tomography
DMINTA	Minimum Air Temperature
DMTA	Maximum Air Temperature
Dtsdam	Total Daily Sunshine Duration
HCl	Hydrochloric Acid
LASSO	Least Absolute Shrinkage and Selection Operator
MAT	Mean Air Temperature
MINDD	Minimum Daylight Duration
MN	Metanephrine
NMN	Normetanephrine
PCC	Pheochromocytoma
PGL	Paraganglioma
PPGL	Pheochromocytoma and Paraganglioma
